# Artificial Serum in Puerperal Septicæmia

**Published:** 1900-04-21

**Authors:** J. Allan Philip


### Hospital Clinics and Medical Progress.

### ARTIFICIAL SERUM IN PUERPERAL SEPTICÆMIA.

By J. Allan Philip, M.A., M.D.

The curative treatment of this formidable disease has by no means kept pace with the preventive treatment, which has succeeded in banishing it from the wards of our lying-in hospitals, although, owing to circumstances not always under the control of the general practitioner, it is still too often seen in private practice. In outlying districts, where the midwife is often the only "skilled" attendant at the bedside, puerperal fever is not uncommon, and, as Dr. Cullingworth pointed out a few years ago, it is responsible for a mortality of over 4 per 1,000 births in some districts in Wales, whilst even in certain metropolitan districts, chiefly occupied by the well-to-do or the fairly well-to-do, it is unduly prevalent. Still, we know that by taking certain precautions puerperal septicæmia will not occur. But when it has once attacked a patient we are poorly equipped to cope with it. By the use of strong doses of quinine, by intra-uterine antiseptic douches, by leeching, poulticing, cool baths, &c., many cases have been cured, but a *foudroyant* case of puerperal fever, unless treated very early, is apt to end fatally. The anti-streptococcus serum has been tried, but has been unfavourably reported on, and I am not aware that any other antitoxin has succeeded. Seeing that the treatment hitherto used has too often disappointed us, I have the less hesitation in bringing under the notice of the profession a remedial agency which, in the few cases in which I have had the opportunity of testing it, has proved so uniformly and rapidly successful that I feel bound to strongly recommend it being tried more extensively. The treatment is simple, and consists of the intra-cellular injection of a saline solution; the one which I have used containing 15 per 1,000 of sodium chloride, and of sodium sulphate. It has been preceded by leeching, but I am disposed to dispense with this in future, as the results I have witnessed are very disproportionate to those of leeching alone in other cases of abdominal or pelvic inflammation, septic or otherwise. After one injection the temperature falls rapidly, and the patient experiences a sensation of well being that is very striking. The temperature may be confidently expected to be 100 deg. or lower within 12 hours, and if it begins to rise again this is at once checked by a second injection. I have not had occasion to repeat it more than once. With the fall of temperature pain rapidly decreases, vomiting, if present, ceases, and natural sleep returns. I have hitherto used only about six ounces of artificial serum at one time, injected into the loose tissue in the front of the abdomen by means of an antitoxin syringe. The syringe is not absolutely necessary, because the fluid spreads freely under the skin, and sufficient pressure to effect this may be obtained by gravitation from a wide-mouthed bottle held a few feet above the patient. The bottle is furnished with two tubes, a curved one for admitting air, and another, connected by indiarubber tubing to a hollow needle, for conveying the fluid. The bottle is inverted for use.

In the last case treated by me the symptoms were very acute, and the result would probably have been a fatal one under any treatment previously known to me. The labour was normal, and the patient's condition afterwards was satisfactory, except for retention of urine and prolonged after-pains, with rather abundant formation of clots. She was syringed twice daily with boric acid solution, and the first unfavourable symptom was a slight rigor on the sixth day, but unaccompanied by fever. Next day there was a slight rise of temperature, and this slowly increased. The uterus was washed out with bichloride solution, and quinine given. During the tenth night a severe and prolonged rigor took place, followed by profuse sweating, and the patient's condition had become critical. I applied half-a dozen leeches, followed by poultices, which abstracted a very fair amount of blood, and I gave her an injection of six ounces of artificial serum. She speedily experienced a sensation of relief, and in the evening the temperature from being over 103 deg. had fallen to 100·5 deg. Next morning it was normal, but the following day it began to rise, and I repeated the injection, which checked the rise. From that time convalescence was uninterrupted. In this case the most likely cause of the sepsis was a prolapsed and chronically congested or inflamed cervix. The local signs were a hot, swollen, and tender vagina, and a tumid and tender uterus, with a sanguinolent discharge, which, curiously enough, did not become at all offensive till improvement had set in.

In another case the symptoms were less acute, and a high oscillating temperature was the most striking one. Local pain, tenderness, &c., were very slight, and the only indication of mischief was fulness and some tenderness on the right of the uterus. Symptoms of involvement of the peritoneum appeared—irritable stomach, tympanites, constipation, &c.—and as quinine, hot vaginal douches, intra-uterine irrigation, and poultices had not effected a cure, I applied six leeches, one of which alighted on a small vein and produced very copious bleeding. At the same time I injected six ounces of the artificial serum, and next day the temperature was normal. I repeated the injection two days after, and a speedy recovery took place.

In considering the *modus operandi* of the artificial serum it is to be noticed th[illegible] two forms of treatment act in a somewhat similar wa[illegible] namely, the use of cold baths in fever, and that of an[illegible] toxins. In all a rapid improvement takes place, proba[illegible]ly due to the arrest of production of toxins in the blood, and it may be that the introduction into the circulation of several ounces of an inorganic fluid acts by destroying the vitality of the septic germs. If this is the case, it would appear that the septic germs possess a rather unstable vitality, and that the severity of the constitutional disturbance is mainly due to the large extent of surface infected by them. I employed the artificial serum first to counteract the lowering effect of the depletion, but the very speedy recovery induced me to continue it as a remedial agency, and although its seeming inadequacy in a grave disease like puerperal septicæmia may strike others, I think it is well worth trying.

I may mention as an interesting fact bearing on the question of licensed midwives, that in this town

(Boulogne-sur-Mer), with an average annual birth-rate of 1,330, the deaths from *affections puerperales*, which means mainly puerperal fever, were for the ten years, ending 1897, 3 3 per annum, or about 2·4 per 1,000. *Sage femmes* do the bulk of the midwifery amongst the lower classes, and smaller tradespeople, and have been trained for a year at a medical school, where they are very thoroughly taught their work. They use antiseptics, usually bichloride, as a matter of routine. In 1897 no case of puerperal fever was notified (this is compulsory).

